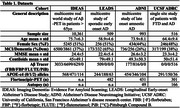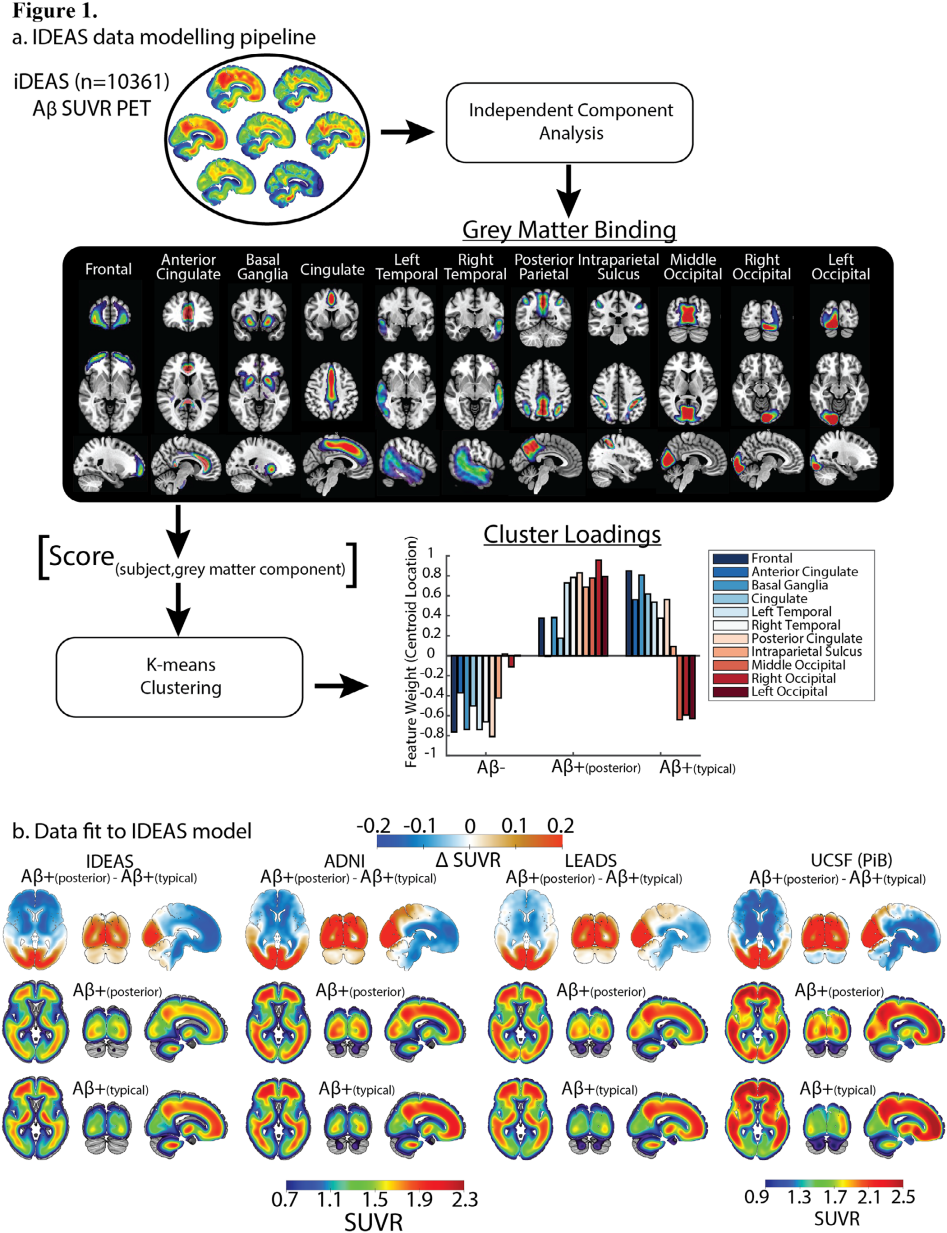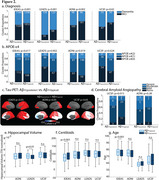# Robust associations with posterior predominant amyloid PET binding

**DOI:** 10.1002/alz70862_110218

**Published:** 2025-12-23

**Authors:** Joseph Giorgio, Ganna Blazhenets, Jhony A. Mejía‐Perez, Daniel R. Schonhaut, Susan M. Landau, Stefania Pezzoli, Jennifer S. Yokoyama, Maria C. Carrillo, Lea T. Grinberg, William W. Seeley, Salvatore Spina, Liana G. Apostolova, Brad C. Dickerson, William J. Jagust, Gil D. Rabinovici, Renaud La Joie

**Affiliations:** ^1^ University of California, Berkeley, Berkeley, CA USA; ^2^ The University of Newcastle, Callaghan, NSW Australia; ^3^ Memory and Aging Center, Weill Institute for Neurosciences, University of California San Francisco, San Francisco, CA USA; ^4^ University of California, San Francisco, San Francisco, CA USA; ^5^ Neuroscience Department, University of California, Berkeley, Berkeley, CA USA; ^6^ Global Brain Health Institute (GBHI), University of California San Francisco (UCSF); & Trinity College Dublin, San Francisco, CA USA; ^7^ Alzheimer's Association, Chicago, IL USA; ^8^ Memory and Aging Center, UCSF Weill Institute for Neurosciences, University of California, San Francisco, San Francisco, CA USA; ^9^ Department of Neurology, Memory and Aging Center, University of California San Francisco, San Francisco, CA USA; ^10^ Indiana Alzheimer's Disease Research Center, Indiana University School of Medicine, Indianapolis, IN USA; ^11^ Frontotemporal Disorders Unit and Massachusetts Alzheimer’s Disease Research Center, Department of Neurology, Massachusetts General Hospital and Harvard Medical School, Boston, MA USA; ^12^ Memory and Aging Center, Weill Institute for Neurosciences, University of California, San Francisco (UCSF), San Francisco, CA USA

## Abstract

**Background:**

The significance of the regional distribution of Aβ‐PET signal is not well established. We used data‐driven approaches to identify Aβ‐PET patterns and their clinicopathological associations in four independent cohorts (12,379 cognitively impaired patients).

**Methods:**

We analysed multi‐tracer template‐space Aβ‐PET SUVR images to assign clinically impaired participants (MCI/Dementia) into groups based on their topography of Aβ‐PET binding. Using the IDEAS cohort, we applied independent component analysis to extract grey matter (GM) components of cortical and subcortical binding we then clustered patients by their score from these GM components using k‐means clustering. We then fit data from three independent cohorts to this model assessing associations between Aβ‐PET patterns and clinical impairment, APOE‐ε4, tau‐PET, and neuropathology (Table 1).

**Results:**

We retained 11 GM components of Aβ‐PET and following k‐means clustering uncovered three clusters of participants in IDEAS (Figure 1a). One cluster had low GM Aβ‐PET binding (i.e., Aβ‐, *n* = 4729, mean CL=2), while the other two were Aβ+ with differences along a posterior‐anterior gradient: Aβ+_(posterior)_ (*n* = 2484, mean CL=76), with predominant occipital binding, and Aβ+_(typical)_ (*n* = 3148, mean CL=86). Applying new data to this model replicated this gradient in each independent dataset (Figure 1b). Contrasting clinical variables for the two Aβ+ clusters showed Aβ+_(posterior)_ patients were more clinically impaired than Aβ+_(typical)_ patients, had a lower proportion of APOE‐ε4 carriership, and had higher posterior tau‐PET (Figure 2a‐c). At autopsy, the Aβ+_(posterior)_ cluster presented with more severe cerebral amyloid angiopathy (CAA) (Figure 2d), but similar Thal and CERAD staging. There were no reliable differences in CL, age, and hippocampal volume (Figure 2e‐g).

**Conclusions:**

We reliably assign patients into an Aβ+_(posterior)_ PET binding group uncovering consistent associations that suggests occipital regions should not be neglected in the appraisal of Aβ‐PET. Specifically, occipital Aβ+ PET binding may be a marker of CAA and more severe cognitive impairment.